# Initial preclinical investigation of aclarubicin treatment in primary cutaneous T-cell lymphoma cells

**DOI:** 10.1016/j.xjidi.2026.100495

**Published:** 2026-05-30

**Authors:** Fenna J. de Bie, Morgann D. Dettingmeijer, Sanne de Haan, Willem H. Zoutman, Sabina Y. van der Zanden, Cornelis P. Tensen, Ferenc A. Scheeren, Jacques Neefjes, Maarten H. Vermeer

**Affiliations:** 1 Department of Dermatology, Leiden University Medical Center, Leiden, The Netherlands; 2 ONCODE Institute, Department of Cell and Chemical Biology, Leiden University Medical Center, Leiden, The Netherlands

**Keywords:** Cutaneous T-cell lymphoma (CTCL), Drug development, T cells

## Abstract

Better therapeutic agents are needed in the treatment of advanced cutaneous T-cell lymphomas. Anthracyclines, including doxorubicin, are such drugs, but they are associated with cardiotoxicity that limits treatment. Aclarubicin is an anthracycline that acts by chromatin damage only and does not show any dose-accumulated cardiotoxicity. We compared the cytotoxicity of different anthracyclines that either induce DNA breaks and/or nucleosome eviction on primary ex vivo patient material. Both cutaneous T-cell lymphoma tumor cells and normal CD4 T cells experience cytotoxicity at the same concentration of anthracyclines. Under a 2-hour pulse exposure that mimics the serum pharmacokinetics of these drugs, aclarubicin was less toxic, with an 8–20-fold lower half-maximal inhibitory concentration than those of the other anthracyclines tested, yet it demonstrated superior ex vivo cytotoxicity under T-cell activation. Aclarubicin should be considered for further preclinical and clinical evaluation in cutaneous T-cell lymphoma.

## Introduction

Cutaneous T-cell lymphomas (CTCLs) are a rare and heterogenous group of non-Hodgkin’s lymphomas of skin-homing T cells that primarily affect the skin. The most common type of CTCL is mycosis fungoides (MF), accounting for 62% of CTCL cases (Willemze et al, 2019). MF lesions present as patches or plaques and can develop into tumors and involve peripheral blood, lymph nodes, and viscera (Girardi et al, 2004). Early-stage MF has a median overall survival of 15.8–35.5 years (Garcia-Diaz et al, 2021) and is treated with skin-directed therapies (Latzka et al, 2023), and advanced-stage MF (IIB and higher) has a 5-year survival of 23–62% (Mourad and Gniadecki, 2020) and often requires systemic treatment (Willemze et al, 2025).

Sézary syndrome (SS) is a rare and aggressive type of CTCL characterized by pruritic erythroderma and the presence of tumor cells in the skin, lymph nodes, and blood. Patients with SS have a poor prognosis, with a 5-year survival rate of around 30–40% (Willemze et al, 2019).

Currently, the only curative option for patients with SS and MF is an allogenic hematopoietic stem cell transplantation (de Masson et al, 2023). However, transplant-related morbidity from side effects, including graft-versus-host disease, infections, and death, has limited its use (Virmani et al, 2015). Given that allogenic hematopoietic stem cell transplantation is not suitable for all patients, numerous alternative therapeutic strategies have emerged. In patients with SS and those with advanced stages of MF, durable remission is uncommon, and therefore, patients receive consecutive treatments until loss of response or intolerability is reached. Current systemic therapies utilized for patients with (advanced-stage) MF/SS include retinoids (such as bexarotene), immunomodulatory modalities (such as IFN-α combined with psoralen plus UVA therapy), histone deacetylase inhibitors (such as panobinostat), and mAbs (such as brentuximab vedotin and mogamulizumab) (Dulmage et al, 2018, Scarisbrick et al, 2021). However, these systemic therapies have toxicity and a limited effect and yield only a 23.3–52.5% 5-year survival (Mourad and Gniadecki, 2020).

This emphasizes the need for new treatments for MF/SS. Anthracyclines, such as doxorubicin (Doxo) and its analogs, are widely used in the treatment of various hematologic malignancies and solid tumors (Sritharan and Sivalingam, 2021). However, the use of anthracyclines is limited by side effects, in particular dose-dependent irreversible cardiotoxicity (Lotrionte et al, 2013). Doxo and daunorubicin (Dauno) induce DNA damage by poisoning topoisomerase IIα, thus generating DNA double-stranded breaks, but also cause chromatin damage by inducing histone eviction (Pang et al, 2013, Tewey et al, 1984). Chromatin damage is the dominant cytotoxic activity of anthracyclines (Qiao et al, 2020, 2024).

It was shown that the ability to cause DNA double-stranded breaks is related to the side effect of cardiotoxicity in anthracycline use (Qiao et al, 2020). Aclarubicin (Acla) and N,N-dimethyldoxorubicin (Dime-Doxo), a detoxified version of Doxo, are both capable of evicting histones but do not induce DNA double-stranded breaks (Qiao et al, 2020). They are also poor substrates for the MDR1 drug exporter (van Gelder et al, 2024). In contrast to Doxo or Dauno, Acla and Dime-Doxo do not induce DNA strand breaks and therefore do not promote secondary tumor carcinogenesis (Qiao et al, 2020). In addition, they do not show any accumulated cardiotoxicity in mouse models and, for Acla, in humans (Qiao et al, 2020, 2024). Specifically, Acla has been used successfully in combination with granulocyte colony-stimulating factor and low-dose cytarabine (ie, cytarabine, aclarubicin, and granulocyte colony-stimulating factor) for the treatment of acute myeloid leukemia (AML) and myelodysplastic syndrome in Japan and China (Liu et al, 2015, Wei et al, 2011). A total of 33–60% of patients achieved a complete response after cytarabine, aclarubicin, and granulocyte colony-stimulating factor treatment (Wei et al, 2011). A recent study compared the second-line therapies after idarubicin (a cardiotoxic anthracycline) combined with cytarabine treatment, which showed a 74% overall complete remission rate for patients treated with cytarabine, aclarubicin, and granulocyte colony-stimulating factor, whereas other treatments showed a complete remission of only 35–37% (Qiao et al, 2024).

In this study, we investigated the effect of using these 4 anthracyclines (Acla, Doxo, Dauno, and Dime-Doxo) as single drugs on MF/SS cells to determine whether treatment with anthracycline variants that only cause chromatin damage and lack various side effects would be an alternative treatment option. When MF/SS stem cells reside in the bone marrow or thymus, anthracycline treatment may eliminate these cells and clear the patient of the tumor, resulting in a full cure.

## Results

### Anthracyclines show cytotoxicity in CTCL cell lines

**Figure 1:**
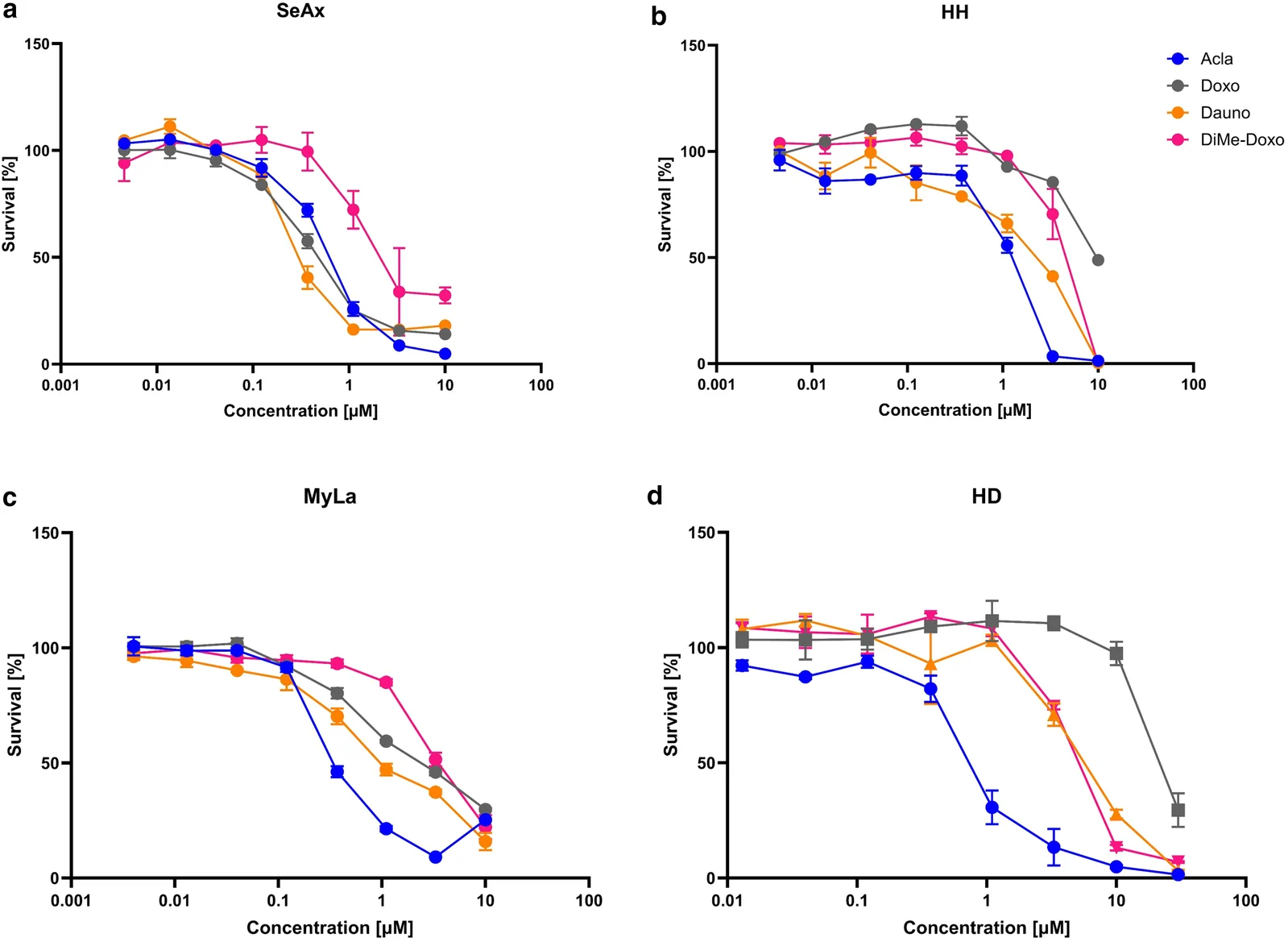
**Drug dose–response experiments on cell lines with anthracyclines.** (**a)** SeAx cells. (**b)** HH cells. (**c)** MyLa cells. (**d)** HD CD4+ T cells were treated for 2 hours with the indicated drug concentrations before drugs were removed to approach the serum pharmacokinetics of these drugs. Seventy-two hours after drug treatment, survival is relative to the untreated control (set as 100%). This is a representative of 3 biological replicates, n = 3 technical replicates with the standard error of the mean. IC_50_ for each drug is indicated in Table 1. On cell lines, the 4 different treatments show no significant differences when using a 1-way ANOVA. HD, healthy donor; IC_50_, half-maximal inhibitory concentration.

To assess the cytotoxic effect of the anthracyclines Doxo and Dauno, which combine DNA damage and chromatin damage, and Acla and Dime-Doxo, which only act by chromatin damage, we performed cell survival assays using CTCL cell lines SeAx, HH, and MyLa. A CellTiter-Blue assay was used as a readout for cytotoxicity. The dose–response data suggest that SeAx cells are sensitive to the different anthracyclines, with a half-maximal inhibitory concentration (IC_50_) of around 0.3 μM (Dime-Doxo is around 3-fold higher than the other 3 anthracyclines), whereas the IC_50_ for cell line HH was 1 μM (Acla and Dauno) versus 5–10 μM for Doxo and Dime-Doxo (Figure 1 and Table 1). The IC_50_ for MyLa cells was 0.1–1 μM for 3 anthracyclines and 4–7 μM for Dime-Doxo. The IC_50_ of healthy donor CD4+ T cells was 1 μM for Acla, 10–20 μM for Doxo, and 3–6 μM for Dauno and Dime-Doxo.

**Table 1:** IC_50_ Calculated for All Cell Line and Patient Experiments Displayed in μM

Sample	Aclarubicin	Doxorubicin	Daunorubicin	Dimethyldoxorubicin
HH cell line rep 1	0.1881	2.56	1.432	0.5402
HH cell line rep 2	1.043	4.084	8.305	ND
HH cell line rep 3	1.152	9.423	1.948	3.743
SeAx cell line rep 1	0.5716	0.9471	0.2449	N/A
SeAx cell line rep 2	0.292	0.5186	0.1029	4.698
SeAx cell line rep 3	0.5241	0.4687	0.2818	2.578
MyLa cell line rep 1	0.2872	0.961	0.8891	6.432
MyLa cell line rep 2	0.1478	0.6865	0.1310	3.857
MyLa cell line rep 3	0.3132	0.2307	0.4009	4.520
Patient 1	1.016	33.61	3.149	7.882
Patient 2	4.93	28.1	10.38	ND
Patient 3	2.226	14.23	5.993	17.21
Patient 4	2.013	19.46	8.131	18.26
Patient 5	1.689	26.1	11.54	20.74
HD 1	0.7012	20.91	3.864	3.363
HD 2	0.9940	10.38	4.201	3.734
HD 3	0.8019	12.62	5.441	4.088
HD 4	1.136	14.19	3.272	5.402

Abbreviations: HD, healthy donor; IC_50_, half-maximal inhibitory concentration; N/A, not available; ND, not detectable.

### Under activated ex vivo conditions, Acla is the most potent anthracycline drug across primary CD4+ T cells from 5 patients with CTCL

**Figure 2:**
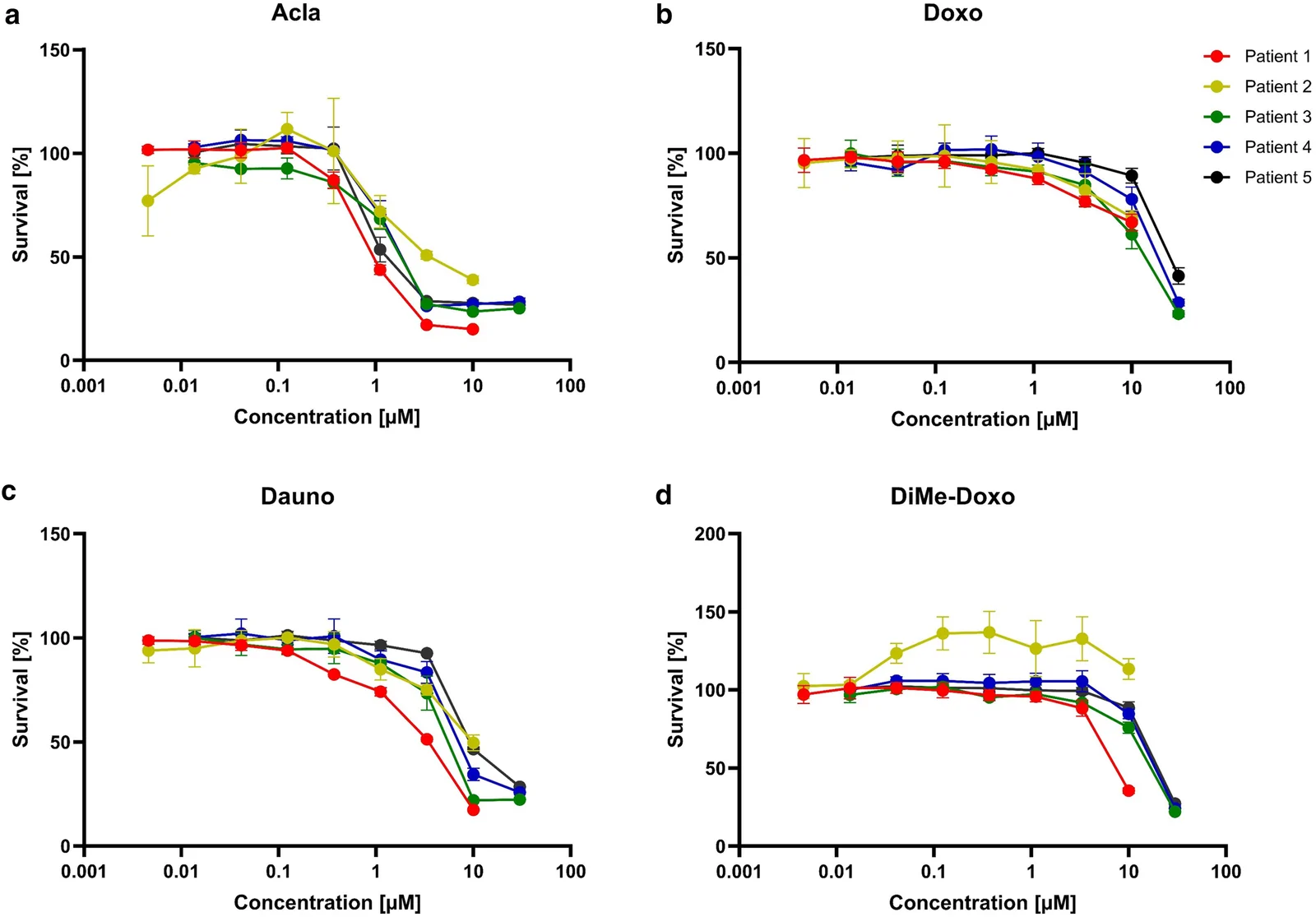
**Drug dose–response experiments of 5 patients treated with 4 different anthracyclines.** (**a)** Survival relative to untreated control (set as 100%) of CD4+ T cells (both tumor and normal) of 5 patients with CTCL 72 hours after a 2-hour exposure to an anthracycline at different indicated concentrations. (**a**) Acla, **(b)** Doxo, (**c)** Dauno, and (**d)** DiMe-Doxo. n = 3 technical replicates with the standard error of the mean. Acla, aclarubicin; CTCL, cutaneous T-cell lymphoma; Dauno, daunorubicin; Dime-Doxo, dimethyldoxorubicin; Doxo, doxorubicin.

**Figure 3:**
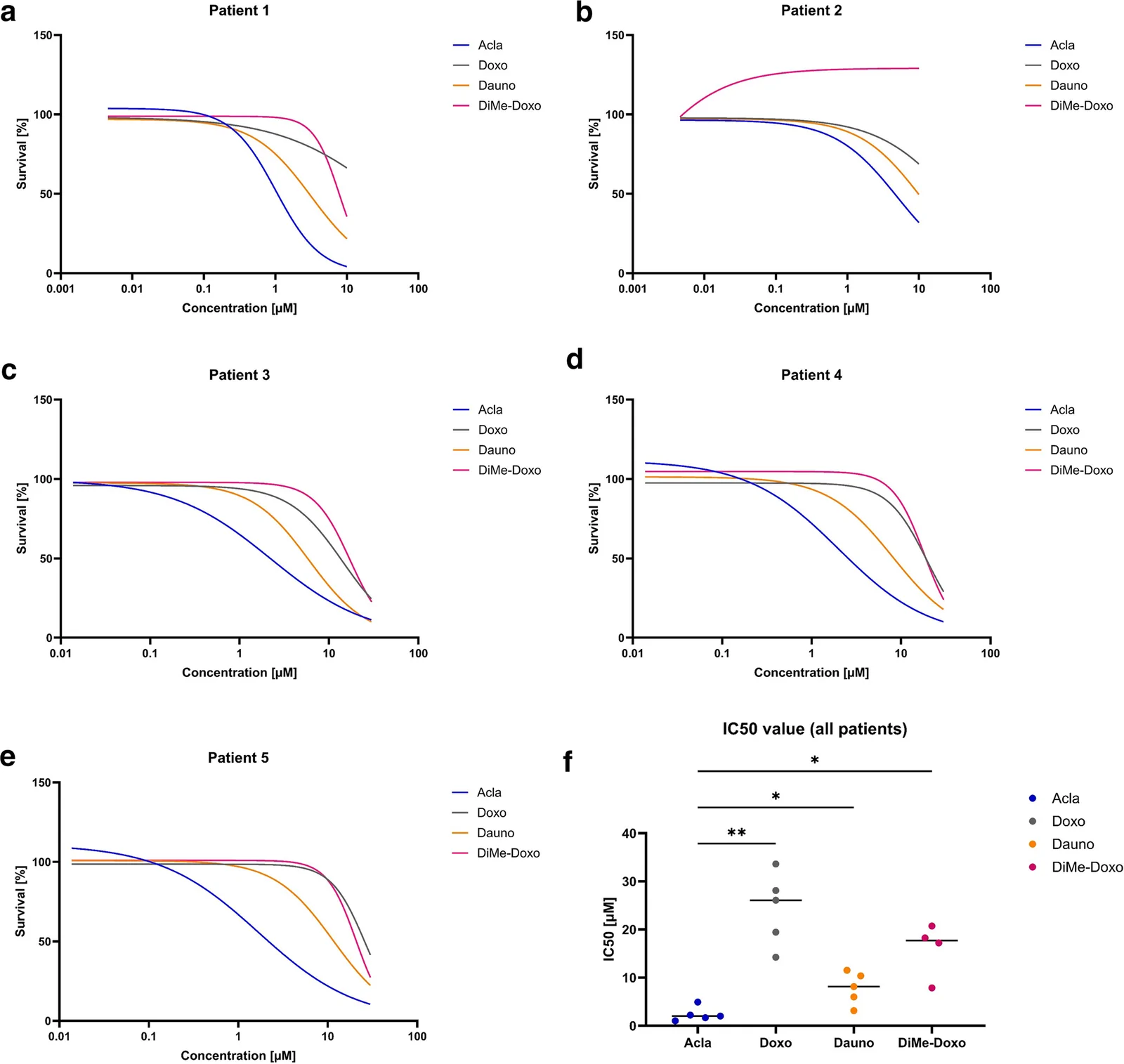
**Drug dose–response experiments of 5 patients treated with 4 different anthracyclines for 2 hours, 72 hours postdrug treatment survival to the untreated control (set at 100%).** (**a)** Dose–response curve of patient 1 showing Acla, Doxo, Dauno, and DiMe-Doxo. (**b)** Dose–response curve of patient 2. (**c)** Dose–response curve of patient 3. (**d)** Dose–response curve of patient 4. (**e)** Dose–response curve of patient 5. (**f)** All IC_50_ of the 5 patients with CTCL. Data were analyzed with 1-way ANOVA with multiple comparisons: ∗*P* < .05 and ∗∗*P* < .01. CTCL, cutaneous T-cell lymphoma; Dauno, daunorubicin; Dime-Doxo, dimethyldoxorubicin; Doxo, doxorubicin; IC_50_, half-maximal inhibitory concentration.

In cell lines, all 4 anthracyclines are cytotoxic; the IC_50_ (Table 1) varied between 0.1 and 9.4 μM. The IC_50_ for Acla is 0.2, 0.3, and 1.0 μM for MyLa, SeAx, and HH cells, respectively. These are established CTCL cell lines. To assess relevant patient-specific cytotoxicity of the different anthracyclines, primary patient CD4+ T lymphocytes were used in an ex vivo dose–response survival assay. Primary CD4+ T cells were activated with 10 U/ml IL-2; 1:10,000 anti-CD3; and 0.2 μg/ml anti-CD28 to sustain viability and mimic T-cell engagement, which affects drug sensitivity. These primary CD4+ T lymphocytes are a mix containing both normal CD4+ T cells and tumor cells; the tumor cell percentage for each patient is shown in Table 2. Again, the cells were exposed to the drugs for only 2 hours and further cultured for 72 hours before analysis. Overall, Acla showed superior cytotoxic effects compared with Doxo, Dauno, and Dime-Doxo in all patients (Figures 2 and 3a–e). The IC_50_ for Acla varied between 1.0 and 4.93 μM among the 5 patients (Table 1). This superior cytotoxicity is further illustrated by drug dose–response curves of all 4 anthracyclines within 1 representative patient (Figure 3a–e). When comparing the IC_50_ of all patients (Figure 3f), Acla demonstrates considerably more potent cytotoxicity, as indicated by a significantly lower IC_50_ than Doxo, Dauno, and Dime-Doxo.

**Table 2:** Patient (n = 5) Characteristics at Blood Sampling

ID	Age at Blood Sampling	Diagnosis	Tumor Percentage^1^	Treatment at Blood Sampling
Patient 1	78	MF IVa	60.0%	Topical corticosteroids
Patient 2	63	SS	40.5%	UVB, prednisone, retinoid
Patient 3	65	SS	84.6%	MTX, prednisone, UVB
Patient 4	68	SS	35.5%	Topical corticosteroids, MTX
Patient 5	72	SS	33.6%	Topical corticosteroids

Abbreviations: ID, identification; MF, mycosis fungoides; MTX, methotrexate; SS, Sézary syndrome.

1 Tumor percentage is the percentage of tumor cells within the CD4+ T-cell population as determined by multiparameter flow analysis.

### No therapeutic window between CTCL and healthy CD4+ T cells

**Figure 4:**
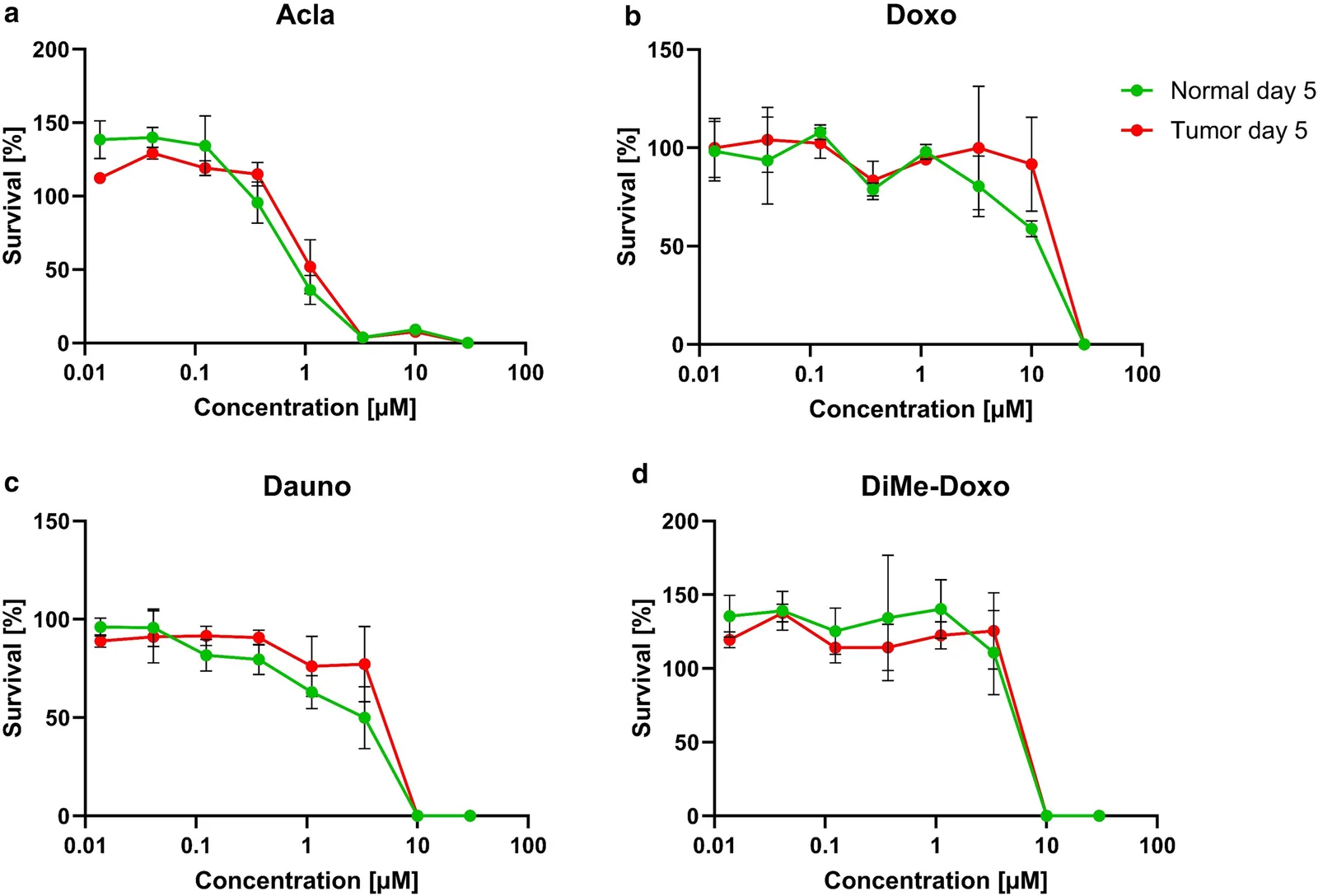
**Relative survival of tumor and normal CD4+ T cells of 1 representative patient 72 hours after a 2-hour treatment with 4 different anthracyclines.** (**a)** Acla. (**b)** Dauno. **(c)** Doxo. (**d)** DiMe-Doxo. Experiment was performed in technical triplicates. Shown is mean ± SD. Acla, aclarubicin; Dauno, daunorubicin; Dime-Doxo, dimethyldoxorubicin; Doxo, doxorubicin.

**Figure 5:**
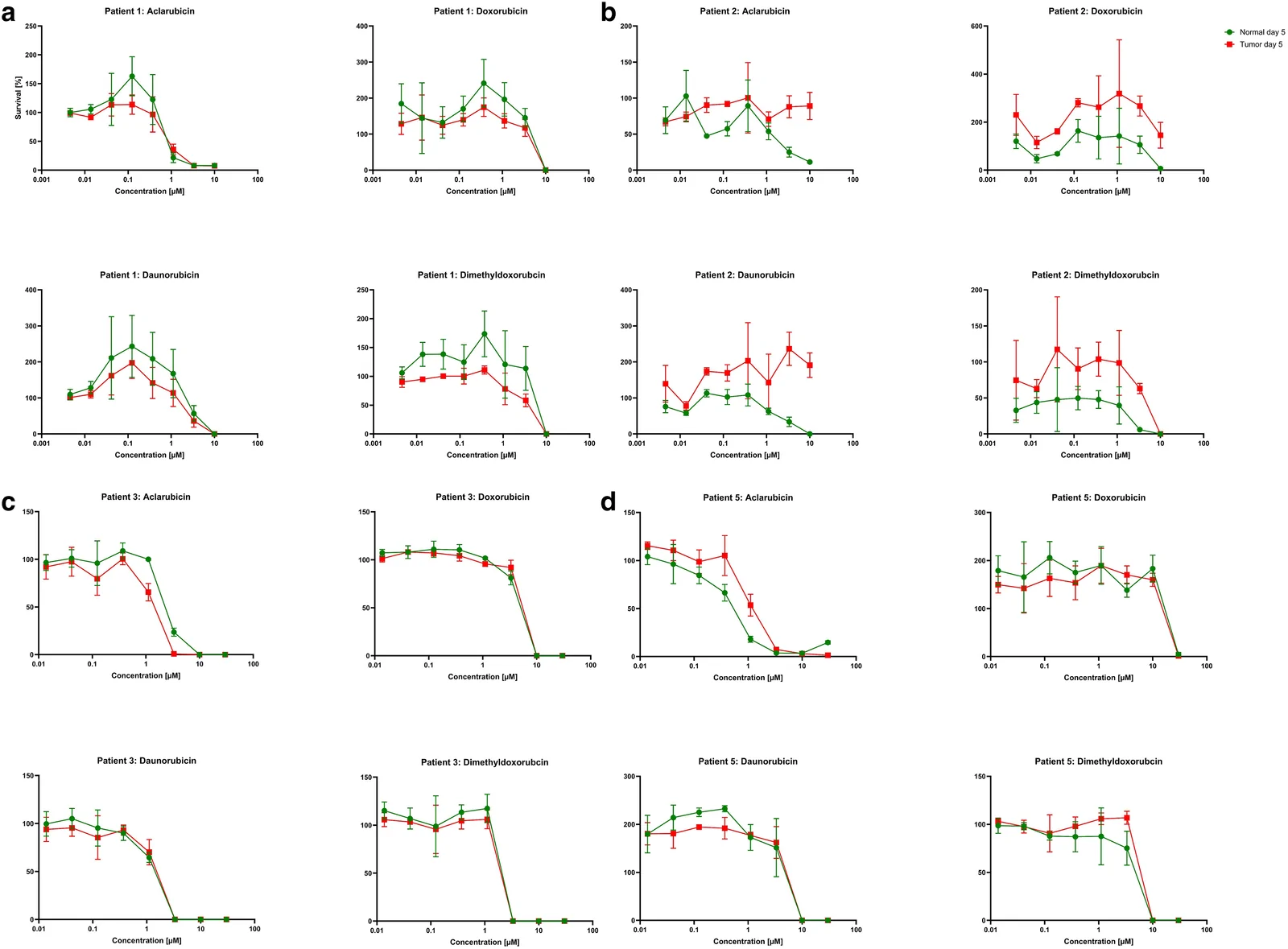
**Relative survival of tumor and normal CD4+ T cells 72 hours after a 2-hour treatment with 4 different anthracyclines.** (**a**) Patient 1. **(b)** Patient 2. (**c)** Patient 3. (**d)** Patient 5. Experiment was performed in technical triplicates. Shown is mean ± SD.

To evaluate whether sensitivity to anthracycline treatment differs between normal and malignant T cells, we exposed a mixture of CTCL and normal healthy CD4+ T cells to different types of anthracyclines and determined cytotoxicity in a dose–response experiment. The cell mixture was exposed to the study drug for 2 hours, which was then removed to mimic the serum pharmacokinetics of these drugs (Murzyn et al, 2024). Seventy-two hours later, spectral flow analysis was performed to analyze CD4+ T cells with a normal or tumorous phenotype (Table 3) separately. Both CTCL tumor cells and healthy CD4+ T cells have a similar sensitivity to the different drugs (Figures 4a–d and 5). Again, the cytotoxicity of Acla was 8–20 times higher than that of the other anthracyclines tested. These cross-drug comparisons under a 2-hour pulse exposure may reflect differences in drug uptake, efflux, or intrinsic cellular sensitivity, indicating pharmacokinetic and/or actual cytotoxic differences between the compounds.

**Table 3:** Gating Strategy on Live CD4+ T Cells to Distinguish between Tumor Cells and Normal CD4+ T Cells

Patient	Normal CD4+ T Cells	Tumor Cells
1	TCR−cB1+/−/CD2+/CD7+/−	TCR−cB1−CD2−/CD7+
2	FSC+/TCR−cB1+/−/CD2+/CD7+/−	SSC+/TCR−cB1−/CD2−/CD7+
3	TCR−cB1+/−/CD2+/CD7+/−	TCR−cB1+/CD2−/CD7+/−/CD27+/CD45RO+
4	TCR−cB1+/−/CD2+/CD7+/−	TCR−cB1−/CD7−/CD26−/CD45dim
5	TCR−cB1+/−/CD2+/CD7+/−	TCR−cB1+/CD2−/CD7+/−/CD3dim/CD26−

Abbreviations: FSC, forward scatter; SSC, side scatter.

## Discussion

In this study, we show the cytotoxicity of anthracyclines on CTCL cell lines SeAx, MyLa, and HH and on ex vivo CD4+ T cells from patients with CTCL (SS and MF) and healthy donors. We show that the cytotoxicity is not specific to CTCL cells but rather to all the CD4+ T cells present. Acla is an anthracycline that has been used in Europe and is still being used successfully in China and Japan for the treatment of patients with AML. The toxicity is limited, which is why this drug is given to frail patients with AML. In addition, Acla can be given many times owing to the absence of accumulated cardiotoxicity. Acla penetrates solid tissues poorly, and its use is then limited to more “blood-borne tumors” such as MF/SS and AML. Acla has shown superior survival outcomes in patients with AML without side effects such as cardiotoxicity (Qiao et al, 2024). The mechanisms of action of anthracyclines are the poisoning of topoisomerase II, causing DNA damage and histone eviction, leading to chromatin damage (Qiao et al, 2024). However, Acla does not induce DNA double-stranded breaks and acts by chromatin damage only, resulting in fewer side effects. Unlike Doxo or Dauno, Acla is not cardiotoxic, does not induce therapy-induced second tumors, does not induce infertility in mice (Qiao et al, 2020), and does not induce fatigue (Wang et al, 2022). This leads us to believe that Acla can be given to patients as a monotherapy during extended periods, similar to how pegylated Doxo or gemcitabine is currently given to patients with MF/SS. Typical response rates for these therapies are 41–58% for pegylated Doxo (Dummer et al, 2012, Weiner et al, 2024) and 55–68% for gemcitabine (Di Raimondo et al, 2022, Duvic et al, 2006, Weiner et al, 2024). It could also replace Doxo in combination therapies, including CHOP (cyclophosphamide, hydroxydaunorubicin [Doxo], oncovin [vincristine], and prednisone), resulting in better, less toxic treatments.

The cytotoxicity of the 4 anthracyclines does not differ between the tumor cells and the healthy cells present in the CD4+ T-cell population, leading to consistent results, although our samples included different blood tumor burdens (33–85% of CD4+ T cells) (Table 2). All 5 patients were differently pretreated; owing to the small cohort size, we cannot draw any conclusions on the influences of other treatments on the anthracycline-induced toxicity. We observed that Acla was 8–20-fold more potent than the other anthracyclines tested, and this was consistent across all 5 patients. These results suggest that the anthracycline Acla could become part of the treatment protocol for patients with MF/SS.

Currently, allogenic hematopoietic stem cell transplantation is the only curative option for patients with advanced stages of MF and SS (de Masson et al, 2023). However, these patients are typically older and have considerable comorbidities, making them often ineligible for this treatment (Novelli et al, 2019). Alternative treatments are (poly)chemotherapy and, more recently, targeted therapies such as brentuximab vedotin and mogamulizumab (Scarisbrick et al, 2021).

However, these systemic therapies are associated with (cumulative) toxicity, limited efficacy, and short-lived clinical responses, resulting in a 5-year survival rate of only 23.3–52.5% (Mourad and Gniadecki, 2020). Therefore, patients with advanced-stage MF/SS who are not eligible for allogenic hematopoietic stem cell transplantation represent a considerable therapeutic challenge.

Combination therapies in CTCL, such as CHOP for MF/SS and BV-CHP (brentuximab vedotin, cyclophosphamide, Doxo, and prednisone) for exceptional cases of CD30+ disease, include Doxo. These therapies are used in aggressive or refractory cases and are often effective, but their therapeutic effect is often short-lived. On the basis of the results from this study, the question is whether Doxo in CHOP therapy should be replaced by Acla to arrive at less toxic and likely more effective treatment protocols.

Pegylated liposomal Doxo is already in use as a systemic treatment for patients with advanced CTCL; the prospective multicenter study EORTC 21012 showed that 6% of patients experienced a complete response, whereas 35% achieved a partial response (Dummer et al, 2012). In 60% of patients, a reduction of cutaneous manifestations of 50% or greater was observed; 4% of patients suffered an early death due to cardiotoxicity or disease progression (Dummer et al, 2012). An Acla-based therapy may provide a different biodistribution of the drug and perhaps more effective elimination of the tumor stem cells, in a situation similar to its role in AML (Qiao et al, 2024).

Our data show an improved sensitivity of Acla over other anthracyclines, which is comparable to results from preclinical studies of Acla in AML (Qiao et al, 2020). Because all ex vivo assays were performed under T-cell activation, the observed potency and lack of tumor selection may be activation dependent and require confirmation under nonactivated conditions. Combined with the knowledge of Acla as an effective anticancer drug with limited cytotoxicity, it should be considered for the treatment of CTCL, in particular SS and MF. These studies should be initiated to improve the prospects for patients with MF and SS.

## Materials and Methods

### Reagents

Doxo was obtained from Accord Healthcare, Dauno was obtained from Sanofi, and Acla (sc-200160) was purchased from Santa Cruz Biotechnology. N,N-Dime-Doxo was synthesized as described (Qiao et al, 2020).

### Cell line culture

SeAx cells, a gift from K. Kaltoft (Kaltoft et al, 1987), and MyLa cells (95051033, ECACC) were cultured in RPMI 1640 with glutamine medium (Gibco) with 10% FB Essence (VWR International), 100 U/ml penicillin–streptomycin (Thermo Fisher Scientific), and 10 U/ml IL-2 (BD Biosciences). HH cells (CRL-2105, ATCC) were cultured in RPMI 1640 with glutamine medium with 10% FB Essence. Cells were plated at 20,000 cells per well (5000 in the case of MyLa) in 90 μl of their culture medium. Cells were allowed to rest overnight in a 37 °C, 5% carbon dioxide incubator. The next day, cells were treated with different doses of anthracyclines (Doxo, Dauno, Dime-Doxo, and Acla); after 2 hours, the plates were spun down, and the supernatant was removed. Fresh medium was added to the cells, and they were incubated for 72 hours. All conditions were executed in triplicate. Cell lines were routinely tested for the absence of mycoplasma by PCR.

### Patient selection

Five patients were selected from the dermatology outpatient clinic of Leiden University Medical Center in The Netherlands. All patients had been previously diagnosed with SS or MF according to World Health Organization criteria and had circulating tumor cells present in their blood (Willemze et al, 2019). Patient characteristics are summarized in Table 2.

### Primary cell culture

PBMCs were isolated from sodium-heparin anticoagulant blood with Ficoll. CD4+ T-cell enrichment followed, utilizing the Magnetic Cell Separation protocol by Miltenyi Biotec (catalog number 130-096-533). A total of 20,000 CD4+ T cells were plated in 90 μl IMDM medium (Lonza) enriched with 8% FB Essence, 100 U/ml penicillin–streptomycin, 10 U/ml IL-2, 1:10,000 anti-CD3 (1XE, Sanquin), and 0.2 μg/ml anti-CD28 (CD28.2, BioLegend) in a 96-well plate (Corning). Cells rested overnight in a 37 °C, 5% carbon dioxide incubator. The next day, cells were treated with different doses of anthracyclines (Doxo, Dauno, Dime-Doxo, and Acla); after 2 hours, plates were spun down, and supernatant was removed. Fresh medium was added to the cells, and cells were incubated for 72 hours. All conditions were executed in triplicate.

### CellTiter-Blue assay

Seventy-two hours after treatment, 10% of CellTiter-Blue (Promega) was added to each well. Fluorescence was measured with a SpectraMax iD3 plate reader (Molecular Devices; excitation 550 nm, emission 590 nm) immediately after adding CellTiter-Blue reagents and after 4 hours of incubation. Relative survival was normalized to that of the untreated control and corrected for background signals.

### Flow cytometry

After CellTiter-Blue measurement, the plates were spun down, and the supernatant was removed. A surface antibody cocktail was added with the following antibodies: CD2 (clone S5.2, BD Biosciences), CD3 (clone SK7, BD Biosciences), CD4 (clone SK3, BD Biosciences), CD7 (clone M-T701, BD Biosciences), CD26 (clone L272, BD Biosciences), CD27 (clone M-T271, BD Biosciences), CD28 (clone CD28.2, BD Biosciences), CD45 (clone HI30, eBioscience), CD45RA (clone HI100, BD Biosciences), CD45RO (clone UCHL1, BD Biosciences), TCR-cβ1 (clone JOVI-1, Immunostep), and 7-amino-actinomycin D (BD Biosciences). After 30 minutes of incubation, PBS (Fresenius Kabi) was added. Samples were measured on a 3-laser Aurora (Cytek Biosciences) and analyzed with Infinicyt software, version 2.1.0a (Cytognos S.L.). Relative survival was normalized to the untreated control for both tumor and healthy CD4+ T cells.

## Ethics Statement

This study was performed in accordance with the Declaration of Helsinki. Collection of human tissue samples for this study was approved as part of the study protocol. This human study was approved by the Medical Ethics Review Committee of Leiden University Medical Center. All adult participants provided written informed consent to participate in this study.

## Data Availability Statement

The datasets generated during and/or analyzed during this study are available from the corresponding author on reasonable request.

## ORCIDs

Fenna J. de Bie: http://orcid.org/0000-0003-3817-8616

Morgann D. Dettingmeijer: http://orcid.org/0009-0009-5778-4903

Sanne de Haan: http://orcid.org/0009-0001-6713-229X

Willem H. Zoutman: http://orcid.org/0000-0003-0085-3647

Sabina Y. van der Zanden: http://orcid.org/0000-0001-5587-1514

Cornelis P. Tensen: http://orcid.org/0000-0002-5325-1888

Ferenc A. Scheeren: http://orcid.org/0000-0002-8304-9023

Jacques Neefjes: http://orcid.org/0000-0001-6763-2211

Maarten H. Vermeer: http://orcid.org/0000-0002-5872-4613

## Conflict of Interest

JN is a minority shareholder of NIHM, which aims to develop aclarubicin for clinical use. The remaining authors state no conflict of interest.
